# miR-590-5p Overexpression Alleviates *β*-Amyloid-Induced Neuron Damage via Targeting Pellino-1

**DOI:** 10.1155/2022/7657995

**Published:** 2022-03-08

**Authors:** Lin Shang, Tao Peng, Xuemei Chen, Zhiyong Yan, Junmin Wang, Xiaoqun Gao, Cheng Chang

**Affiliations:** ^1^Department of Human Anatomy, School of Basic Medicine, Zhengzhou University, China; ^2^Department of Neurology, The First Affiliated Hospital of Zhengzhou University, China

## Abstract

Alzheimer's disease (AD) is one common degenerative disorder. However, the effects of miR-590-5p on AD and the mechanism on modulation of AD development were unclear. In this study, the miR-590-5p level in AD patients at mild, moderate, and severe stage as well as APP/PS1 transgenic mice was detected by qRT-PCR. The relationship of miR-590-5p and pellino-1 (PELI1) was identified by double luciferase reporter gene assay. Afterwards, both BV-2 and HT22 cells were exposed to *β*-amyloid (A*β*) peptides to mimic AD cell model. Then, the roles of miR-590-5p upregulation or PELI1 silence in cell proliferation and apoptosis were explored by CCK-8 assay and TUNEL assay, and the expression of apoptosis-related proteins was detected by western blotting. Furthermore, the involvements of the downstream Traf3/MAPK P38 pathway with the roles of miR-590-5p in AD were measured by western blotting. Our results showed that knockdown of miR-590-5p was found in AD patients, mice model, and A*β*-induced cell model. Notably, PELI1 was proved as a target gene of miR-590-5p. miR-590-5p mimic or PELI1 silence significantly promoted cell proliferation and inhibited cell apoptosis, as well as suppressed the activation of Traf3/MAPK P38 pathway both in A*β*-induced BV-2 and HT22 cells. The effects of PELI1 overexpression on cell proliferation, apoptosis, and Traf3/MAPK P38 pathway were partly abrogated by miR-590-5p mimic both in BV-2 and HT22 cells. In conclusion, miR-590-5p was expressed at lower levels in AD, and miR-590-5p/PELI1 axis might be involved in the progression of AD by the downstream Traf3/MAPK P38 pathway.

## 1. Introduction

Alzheimer's disease (AD) is one highly common degenerative disorder, characterized by several clinical manifestations, such as disorders in activities of daily living and progressive decline of memory and cognitive deterioration [1]. As per the World Health Organization (WHO) statistics, AD was the fifth leading cause affecting human health worldwide in 2016 with high rates of morbidity and mortality as well as great financial cost [2]. AD possesses a number of etiologies, such as accumulation of *β*-amyloid (A*β*) plaques and neurofibrillary tangles, central hyperexcitability, and neuroinflammation [3]. Despite the increased understanding of pathogenesis, current treatment for AD remains a symptom improvement without stopping the progression of disease and altering the ultimate prognosis [4]. Researchers have found that early screening, detection, and diagnosis of AD will allow for early disease intervention at a potentially reversible stage [4, 5]. Thus, searching for effective diagnostic and therapeutic targets for AD is quite essential.

MicroRNAs (miRNAs) are a large type of noncoding small RNA molecules consisting of approximately 22 nucleotides that suppress the expression of target gene through mRNA degradation mediated by the RNA-induced silencing complex in the posttranscriptional level [6]. As an evolutionally conserved group of noncoding RNAs, microRNAs possess tissue- and developmental stage-specific expression and exert vital roles in mediating several cellular processes, such as cell differentiation, proliferation, and apoptosis, thus substantially involved in the pathogenesis of distinct human disorders [7, 8]. Due to the small size, membrane-lipid amphiphilicity, and high solubility, miRNAs are highly mobile and exist in the whole brain and central nervous system [9]. It is for this reason that miRNAs have attracted extensive attention as predictive and diagnostic biomarkers for the occurrence and progression of AD [10]. Nowadays, a large number of miRNAs including miR-101 [11], miR-107 [12], miR-29 [13], and miR-124 [14] are regarded as promising diagnostic biomarkers for AD. Specifically, miR-590-5p is shown to be expressed at lower levels in serum of AD patients than that in healthy subjects based on GSE147232 from GEO database. miR-590-5p has been proved to participate in cancer progression as tumor promoter [15, 16]. However, the precise role and mechanisms of miR-590-5p in AD remain unexplored.

In the current study, the level of miR-590-5p was firstly detected in clinical AD patients with different stage and AD model mice. Then, the effects of miR-590-5p abnormal expression on cell proliferation and apoptosis in A*β*-induced AD cell model were unraveled. Furthermore, the regulation mechanism involving the target gene of miR-590-5p and downstream signaling pathway in AD were explored.

## 2. Materials and Methods

### 2.1. Clinical Sample Collection

Definite AD patients were diagnosed according to the Consortium to Establish a Registry for Alzheimer's Disease (CERAD) criteria [17]. According to the time of suffering AD, patients were divided into 3 groups, the mild stage (1~2 years), moderate stage (2~8 years), and severe stage (8~12 years). Peripheral blood samples from AD patients at mild, moderate, and severe stage (*n* = 15, each stage) were collected at the First Affiliated Hospital of Zhengzhou University from June 2020 to December 2020. Meanwhile, peripheral blood samples from 15 age- and sex-matched healthy volunteers were obtained as normal controls on August 2020. For the blood samples used in this study, prior patient consent and approval were obtained from the patient, and this study was approved by the First Affiliated Hospital of Zhengzhou University.

### 2.2. Animal Sample Collection

The ethics committee of the First Affiliated Hospital of Zhengzhou University gave permission to conduct these animal experiments. Male wild-type mice (*n* = 6) and B6C3-Tg (APPswe, PSEN1dE9)/Nju transgenic mice (6~8 weeks old; ~20 g; *n* = 6) were purchased from the National Resource Center for Mutant Mice (Nanjing, China) and raised in our laboratory. At seven months of age, mice were euthanized, the blood was collected from the heart in each group, and then, the cerebral cortex tissue, hippocampal tissue, and serum were obtained for further experiments.

### 2.3. Cell Culture and Treatment

BV-2 microglial cells and HT22 were purchased and recovered in complete DMEM medium (Thermo, China) before use and cultured with 5% carbon dioxide at 37°C. Both BV-2 and HT22 cells were exposed to 10 *μ*M of A*β* peptides to mimic AD cell model. For analysis of the impacts of miR-590-5p overexpression on AD cell model, miR-590-5p mimic or vector was transiently transfected into A*β*-induced BV-2 and HT22 cells, respectively, using the Lipofectamine™ 2000 transfection reagent (Thermo). In addition, A*β*-induced BV-2 and HT22 cells were, respectively, transfected with PELI1 siRNA (5′-UUCUUGAUCAGGAGAAAACAU-3′) to explore the effects of PELI1 silence on AD cell model. To further explore the relationship of miR-590-5p and PELI1, both BV-2 and HT22 cells were transfected with PELI1 overexpression vector (PELI1 CDS+3′UTR) and/or miR-590-5p mimics, respectively.

### 2.4. Quantitative RT-PCR

The treated cells, serum, and tissues were harvested to obtain total RNA samples by the Invitrogen TRIzol reagent (Thermo). Then, complementary DNA samples were prepared from the isolated RNA based on the QuantiTect Reverse Transcription Kit (QIAGEN). The following quantitative PCR method was finished using the SYBR Premix Ex Taq™ II (Takara). *β*-Actin or U6 was used as the internal standards for PELI1 or miR-93-5p quantitation, respectively. Sequences of primers are all presented in Table 1.

### 2.5. Western Blotting Assay

The treated cells and tissues were lysed using lysis buffer (Beyotime, China) on ice to extract proteins. Then, protein samples underwent resolving on PAGE gel and transferring to PVDF, followed by blocking the membrane and reacting with primary antibody of PELI1 (1 : 1000, Abcam), Traf3 (1 : 1000, Abcam), ERK1/2 (1 : 1000, Abcam), phosphorylated-ERK1/2 (p-ERK1/2) (1 : 1000, Abcam), P38 (1 : 1000, Abcam), p-38 (1 : 1000, Abcam), Bax (1 : 1000, Abcam), Bcl-2 (1 : 1000, Abcam), or GAPDH (1 : 1000, Abcam) at room temperature for 3 h. The second antibody (1 : 3000, Abcam) was then reacted with the membrane, and the expressions of these proteins were observed using enhanced chemiluminescence (Millipore, USA).

### 2.6. Luciferase Activity Assay

The target genes of miR-590-5p were predicted by TargetScan and Starbase databases and then identified by luciferase reporter gene assay. The psiCHECK-2 promoter vector luciferase gene was inserted with mutant 3′UTR of *PELI1* (MUT) and wild-type 3′UTR of *PELI1* (WT) fragments, yielding recombinant plasmids psiCHECK-2-*PELI1*-WT and psiCHECK-2-*PELI1*-MUT. Subsequently, psiCHECK-2-*PELI1*-WT or psiCHECK-2*-PELI1*-MUT, miR-590-5p mimics or NC, and plasmid pRL-TK with Renilla luciferase were cotransfected into 293T cells via Lipofectamine™ 2000 for 24 h. Ultimately, the luciferase activity was measured.

### 2.7. Cell Counting Kit-8 (CCK-8) Assay

The viabilities of cells that underwent the above treatments were evaluated by CCK-8 (Beyotime). Briefly, both BV-2 and HT22 cells were inoculated to the 96-well plates and then treated for another 24, 48, or 72 h, respectively, as above described, which were then reacted with CCK-8 for 2 h. The absorbances at 450 nm (OD_450_) were finally detected by the microplate reader. At least three biological replicates of the CCK-8 assay were performed for evaluating cell viability.

### 2.8. Cell Apoptosis Assay

TUNEL kit (Beyotime) was utilized to detect cell apoptosis. Briefly, both BV-2 and HT22 cells at the logarithmic growth phase underwent the above approaches for 24 h. Next, 4% paraformaldehyde was used to fix cells, and the cells underwent permeabilization and incubation with TUNEL assay for 1 h. Lastly, the cells were observed using inverted microscope.

### 2.9. Cell Immunofluorescence

The expression of PELI1 was also evaluated by cell immunofluorescence. In brief, the cells were fixed in 4% formaldehyde solution for 12 min, blocked with 5% BSA solution, incubated with antibodies targeting PELI1 (1 : 500, Abcam) overnight at 4°C, washed again with PBS solution, and incubated in dark with the secondary antibodies for 1-2 h. Cell nuclei were observed by DAPI staining and then mounted with the ProLong Gold Antifade Reagent (CST). The fluorescence intensities were finally evaluated by observation under fluorescence microscopy.

### 2.10. Statistical Analysis

Quantitative data were analyzed using the SPSS 20.0 software. The differences among various groups were evaluated by *t* test or ANOVA. A *P* value of <0.05 was set as the threshold.

## 3. Results

### 3.1. The Level of miR-590-5p in AD Patients and Mice Model

**Figure 1:**
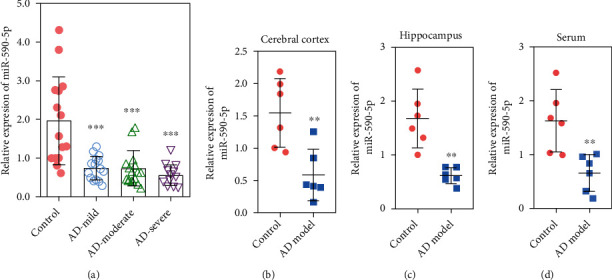
The level of miR-590-5p was lower in AD patients and mice model. (a) The miR-590-5p level in serum of AD patients at mild, moderate, and severe stage as well as healthy volunteers by qPCR (*n* = 15). (b–c) The miR-590-5p level in the cerebral cortex tissue, hippocampal tissue, and serum from wild-type mice (control) and APP/PS1 transgenic mice (AD model) by qPCR (*n* = 6). Each experiment was repeated three times. ^∗∗^*P* < 0.01 and ^∗∗∗^*P* < 0.001 vs. control group.

QPCR assay was applied to analyze the miR-590-5p level in AD patients and mice model. The results showed that the level of miR-590-5p in serum of AD patients at mild, moderate, and severe stage was lower than that in healthy volunteers and no difference on the level of miR-590-5p in mild, moderate, and severe stage of AD patients (*P* < 0.05, Figure 1(a)). Consistently, compared with wild-type mice, the miR-590-5p level was significantly downregulated in the cerebral cortex tissue, hippocampal tissue, and serum from APP/PS1 transgenic mice (*P* < 0.05, Figures 1(b)–1(d)).

### 3.2. PELI1 Was Identified as a Target Gene of miR-590-5p

**Figure 2:**
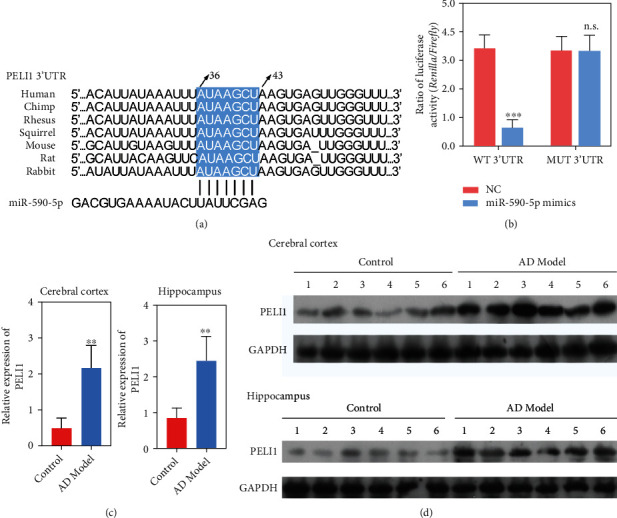
PELI1 was identified as a target gene of miR-590-5p. (a) Prediction of the binding site of miR-590-5p and PELI1 by the TargetScan and Starbase databases. (b) Identification of target regulation of miR-590-5p and PELI1 proved by luciferase reporter system. (c, d) The mRNA and protein levels of PELI1 in the cerebral cortex and hippocampal tissue from wild-type mice (control) and APP/PS1 transgenic mice (AD model) by qPCR and western blotting. Each experiment was repeated three times. ^∗∗^*P* < 0.01 vs. control group.

Through bioinformatics analysis, we predicted that miR-590-5p might directly bind to the *PELI1* gene (Figure 2(a)). Then, the dual luciferase reporter assay found the decreased luciferase activity induced by miR-590-5p mimics in cells with WT, but not in cells with MUT (Figure 2(b)). Moreover, the mRNA and protein levels of PELI1 were higher in the hippocampus and cerebral cortex tissues from APP/PS1 transgenic mice than those in wild-type mice (*P* < 0.05, Figures 2(c) and 2(d)).

### 3.3. Effect of miR-590-5p Overexpression on A*β*-Induced BV-2 and HT22 Cells

**Figure 3:**
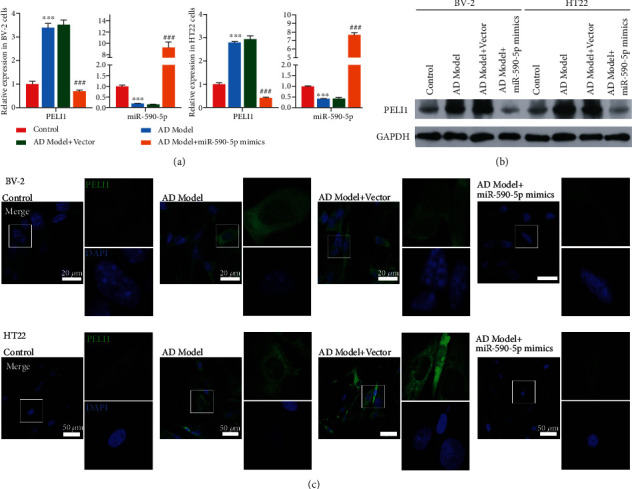
Effect of miR-590-5p overexpression on PEIL1 in A*β*-induced BV-2 and HT22 cells. (a) The mRNA levels of miR-590-5p and PELI1 in BV-2 or HT22 cells treated with A*β* (AD model), A*β*+vector, and A*β*+miR-590-5p mimics, respectively, by qPCR. (b) The protein levels of PELI1 in BV-2 or HT22 cells treated with A*β* (AD model), A*β*+vector, and A*β*+miR-590-5p mimics, respectively, by western blotting. (c) The PELI1 expression in BV-2 or HT22 cells treated with A*β* (AD model), A*β*+vector, and A*β*+miR-590-5p mimics, respectively, by cell immunofluorescence. Each experiment was repeated three times. ^∗∗^*P* < 0.01, ^∗∗∗^*P* < 0.001 vs. control group; ^###^*P* < 0.001 vs. AD model+vector group.

**Figure 4:**
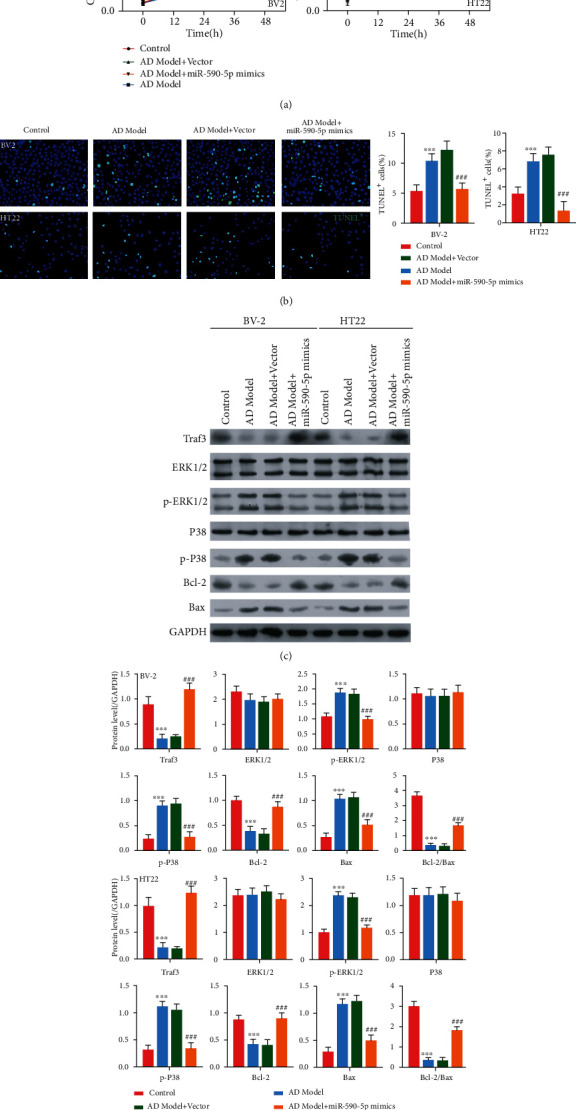
Effect of miR-590-5p overexpression on cell viability, apoptosis, and Traf3/MAPK P38 pathway in A*β*-induced BV-2 and HT22 cells. (a) Cell viability of BV-2 or HT22 cells treated with A*β* (AD model), A*β*+vector, and A*β*+miR-590-5p mimics, respectively, by CCK-8 assay. (b) The cell apoptosis of BV-2 or HT22 cells treated with A*β* (AD model), A*β*+vector, and A*β*+miR-590-5p mimics, respectively, by TUNEL assay (blue, nucleus; green, TUNEL-positive cells). (c, d) The expression of Traf3, phosphorylation levels of ERK1/2 and P38, and apoptosis-related proteins (Bcl-2 and Bax) in BV-2 or HT22 cells treated with A*β* (AD model), A*β*+vector, and A*β*+miR-590-5p mimics, respectively, by western blotting. Each experiment was repeated three times. ^∗∗∗^*P* < 0.001 vs. control group; ^###^*P* < 0.001 vs. AD model+vector group.

We first established AD cell model by A*β* stimulation of BV-2 and HT22 cells. Consistent with AD patients and mice model, the miR-590-5p level in A*β*-induced BV-2 or HT22 was lower than that in control cells (*P* < 0.05, Figure 3(a)). Meanwhile, the PELI1 expression was measured by qPCR, western blotting, and cell immunofluorescence, and the results revealed that both mRNA and protein levels of PELI1 were remarkably increased in A*β*-induced BV-2 or HT22 compared with control cells (*P* < 0.05, Figures 3(a)–3(c)). However, miR-590-5p mimics significantly increased miR-590-5p level and inhibited the expression of PELI1 in A*β*-induced BV-2 cells or HT22 (*P* < 0.05, Figures 3(a)–3(c)). In addition, CCK-8 assay showed that A*β* treatment significantly reduced cell viability both in BV-2 and HT22 cells, while the addition of miR-590-5p mimics partly reversed cell viability (*P* < 0.05, Figure 4(a)). Meanwhile, TUNEL assay revealed that compared with control cells, the TUNEL-positive cells were increased in A*β*-induced BV-2 and HT22 cells, while miR-590-5p mimic reduced the TUNEL-positive cells (Figure 4(b)). Consistently, the expressions of Bax and Bcl-2 were detected by western blotting, and we found that A*β* treatment increased the expression of Bax and inhibited the expression of Bcl-2 both in BV-2 and HT22 cells, while the addition of miR-590-5p mimics partly reversed their expressions (Figure 4(c)). Furthermore, western blotting assay showed that compared with control cells, A*β*-induced BV-2 and HT22 cells exhibited decreased Traf3 expression and increased phosphorylation levels of ERK1/2 and P38, while the addition of miR-590-5p mimics partly reversed their expressions (Figure 4(d)).

### 3.4. Effect of PELI1 Silence on A*β*-Induced BV-2 and HT22 Cells

**Figure 5:**
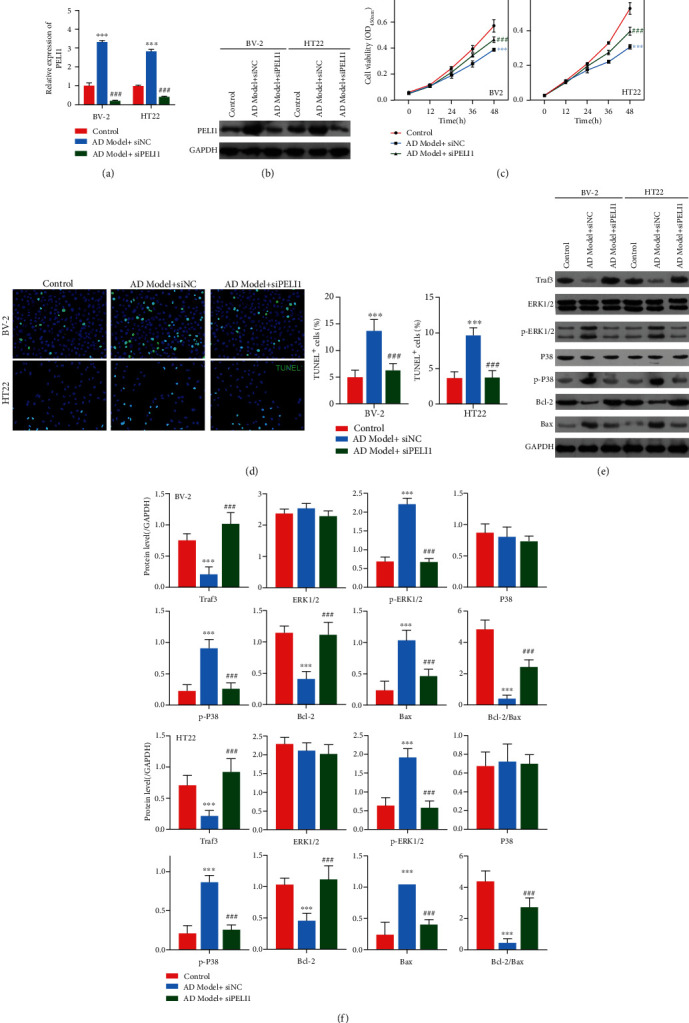
Effect of PELI1 silence on A*β*-induced BV-2 and HT22 cells. (a, b) The mRNA and protein levels of PELI1 in BV-2 or HT22 cells treated with A*β* (AD model), A*β*+siNC (negative control), and A*β*+siPELI1, respectively, by qPCR and western blotting. (c, d) Cell viability of BV-2 or HT22 cells treated with A*β*, A*β*+siNC, and A*β*+siPELI1, respectively, by CCK-8 assay. (e) The cell apoptosis of BV-2 or HT22 cells treated with A*β*, A*β*+siNC, and A*β*+siPELI1, respectively, by TUNEL assay (blue, nucleus; green, TUNEL-positive cells). (f) The expression of Traf3, phosphorylation levels of ERK1/2 and P38, and apoptosis-related proteins (Bcl-2 and Bax) in BV-2 or HT22 cells treated with A*β*, A*β*+siNC, and A*β*+siPELI1, respectively, by western blotting. Each experiment was repeated three times. ^∗∗∗^*P* < 0.001 vs. control group; ^###^*P* < 0.001 vs. AD model group.

To explore the effects of PELI1 silence on AD cell model, A*β*-induced BV-2 and HT22 cells were, respectively, transfected with PELI1 siRNA. The results revealed that both mRNA and protein levels of PELI1 were remarkably increased in A*β*-induced BV-2 or HT22 compared with control cells, while the addition of PELI1 siRNA significantly inhibited the expression of PELI1 in A*β*-induced BV-2 or HT22 (*P* < 0.05, Figures 5(a) and 5(b)). Then, CCK-8 assay showed that in comparison with A*β*-induced BV-2 and HT22 cells, the addition of PELI1 siRNA increased cell viability (*P* < 0.05, Figure 5(c)). Meanwhile, TUNEL assay revealed that compared with AD model cells, PELI1 siRNA reduced the TUNEL-positive cells (Figure 5(d)). Consistently, PELI1 siRNA increased the Bcl-2 expression and decreased the Bax expression both in BV-2 and HT22 cells (Figures 5(e) and 5(f)). Furthermore, compared with AD model cells, cotreatment of PELI1 siRNA with A*β* increased Traf3 expression and decreased phosphorylation levels of ERK1/2 and P38 both in BV-2 and HT22 cells.

### 3.5. Effect of miR-590-5p Overexpression on BV-2 and HT22 Cells Was Regulated by PELI1

**Figure 6:**
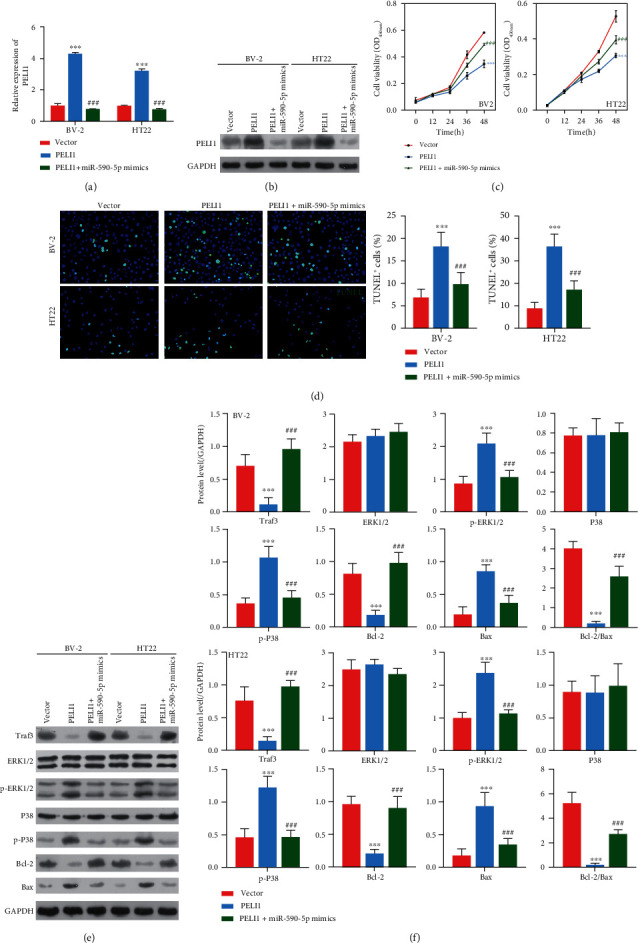
Effect of miR-590-5p silence on BV-2 and HT22 cells was regulated by PELI1. (a, b) The mRNA and protein levels of PELI1 in BV-2 or HT22 cells treated with vector, PELI1 (overexpression of PELI1 CDS+3′UTR), and PELI1 (overexpression of PELI1 CDS+3′UTR)+miR-590-5p mimics, respectively, by qPCR and western blotting. (c, d) Cell viability of BV-2 or HT22 cells treated with vector, PELI1, and PELI1+miR-590-5p mimics, respectively, by CCK-8 assay. (e) The cell apoptosis of BV-2 or HT22 cells treated with vector, PELI1, and PELI1+miR-590-5p mimics, respectively, by TUNEL assay (blue, nucleus; green, TUNEL-positive cells). (f) The expression of Traf3, phosphorylation levels of ERK1/2 and P38, and apoptosis-related proteins (Bcl-2 and Bax) in BV-2 or HT22 cells treated with vector, PELI1, and PELI1+miR-590-5p mimics, respectively, by western blotting. Each experiment was repeated three times. ^∗∗∗^*P* < 0.001 vs. vector group; ^###^*P* < 0.001 vs. PELI1 group.

To further explore the relationship of miR-590-5p and PELI1, both BV-2 and HT22 cells were treated with PELI1 (overexpression of PELI1 CDS+3′UTR) and/or miR-590-5p mimics, respectively. We found that overexpressing PELI1 significantly elevated the mRNA and protein levels of PELI1, while miR-590-5p mimics inhibited the PELI1 expression (*P* < 0.05, Figures 6(a) and 6(b)). Additionally, CCK-8 assay showed that in comparison with control cells, cell viability was inhibited in BV-2 and HT22 cells treated with overexpressing PELI1, whereas the impact of overexpressing PELI1 was subsequently recovered by miR-590-5p mimics (*P* < 0.05, Figure 6(c)). Meanwhile, TUNEL assay revealed that induction of apoptosis by overexpressing PELI1 was also reversed in BV-2 and HT22 cells transfected with miR-590-5p mimics (Figure 6(d)). Furthermore, PELI1 overexpression decreased the expression of Traf3 and Bcl-2, as well as increased the expression of p-ERK1/2, p-P38, and Bax both in BV-2 and HT22 cells, while miR-590-5p mimics partly reversed their expression (Figures 6(e) and 6(f)).

## 4. Discussion

It is widely recognized that miRNAs can decrease its target gene expression by binding the 3′UTR and participate into many key biological processes of disease progression [18]. Previous investigations have demonstrated that the pathogenesis of AD was partly mediated by the multiple microRNAs and the resultant alteration of functional gene expression [19]. It has been reported that miR-590-5p participates in the progression of several cancers. However, whether miR-590-5p exerts vital role in the occurrence and progression of AD remains unclear. The present study showed that miR-590-5p was expressed at lower levels in serum of AD patients when compared with that in serum of healthy people. Importantly, PELI1 was a target gene of miR-590-5p and was inhibited by miR-590-5p. miR-590-5p mimic significantly promoted cell proliferation and reduced cell apoptosis both in A*β*-induced BV-2 and HT22 cells, which could be reversed by PELI1 overexpression. In addition, miR-590-5p inhibited the Traf3/MAPK P38 pathway, which means it plays an antiapoptotic role in AD. Consequently, we speculated that the expression of miR-590-5p in the brain tissue was inhibited during the occurrence and development of AD, and it may have a neuroprotective effect in AD.

As introduced above, microRNAs are critically implicated in multiple diseases, mainly due to their great potency of suppressing target gene expression. Herein, miR-590-5p level was greatly reduced in AD patients at mild, moderate, and severe stage as well as APP/PS1 transgenic mice compared to control group. However, there is no difference on the level of miR-590-5p in mild, moderate, and severe stage of AD patients. The possible reasons are as follows: The abundance of mirR-590-5p in serum is relatively low, which is limited by RNA extraction or detection methods, resulting in excessive detection error; the level of miR-590-5p in serum samples may vary greatly among individuals; the number of sample collected in this study is relatively small, resulting in no statistical difference; in fact, there is little difference in the expression level of miR-590-5p in serum of AD patients at different stages. We will explore the relation between the development stage of AD and expression level of miR-590-5p with a larger sample size in the future research.

Previous reports have shown the functions of miR-590-5p in various diseases through targeting different genes. For example, miR-590-5p level is shown to be dramatically reduced in HepG2 cells, and the overexpression of miR-590-5p is able to inhibit cell proliferation via targeting S100A10 and Wnt signaling pathway by exerting an antitumor role [20]. Similarly, miR-590-5p is proposed to be expressed at lower levels in colorectal cancer, and miR-590-5p upregulation restrains the tumor proliferation and metastasis by inhibiting YAP1 [21]. Another study has also demonstrated that upregulation of miR-590-5p impedes breast cancer progression involving with suppression of cell migration and proliferation via regulating SOX2 [22]. Contradictorily, a study of miRNA expression profile has shown that miR-590-5p is overexpressed in squamous cell carcinoma and functions as the tumor promoter [23]. In addition, miR-590-5p is downregulated in oxidized low density lipoprotein- (ox-LDL-) induced HUVECs, and the overexpression of miR-590-5p inhibits the apoptosis of HUVECs induced by ox-LDL [24]. In this study, we found that miR-590-5p overexpression promoted cell proliferation and induced cell apoptosis in A*β*-induced BV-2 and HT22 cells, which suggested that miR-590-5p exhibited protective effect against cell injury induced by A*β*.

Furthermore, the *PELI1* gene was proved as one target gene of the miR-590-5p by bioinformatics in this study. Subsequently, we verified the direct binding of miR-590-5p with the 3′UTR region of *PELI1* gene through the dual luciferase assay. The targeting role of *PELI1* gene expression by miR-590-5p was further supported by the significant elevation of PELI1 expression in A*β*-induced BV-2 and HT22 cells treated by miR-590-5p mimics. PELI1, as an ubiquitination E3 ligase, plays a role in regulating protein ubiquitination and degradation [25]. It has been shown that the ubiquitin-proteasome system regulates the loss of P-glycoprotein (P-gp) in AD, and inhibition of P-gp ubiquitination hinders P-gp degradation and reduces A*β* level in the brain tissues [26]. In addition, Katayama et al. have revealed that SCFFBX15, as one ubiquitin E3 ligase complex, is able to induce P-gp ubiquitination [27]. In our study, it was disclosed that PELI1 was highly expressed in AD, and the inhibiting effects of miR-590-5p mimic on A*β*-induced BV-2 or HT22 cell injury were partly abrogated PELI1 overexpression. All these observations persuasively proved that miR-590-5p/PELI1 axis was involved in A*β*-induced BV-2 or HT22 cell injury, which might be closely related to abnormal ubiquitination in AD. However, there is a limitation of experimental design, which is lacking of miR-590-5p or PELI1 intervention in AD animal models. The mice need to be raised to 7 months old to establish AD animal models, which make it difficult to supplement the experiment.

Moreover, we explored the downstream signaling pathway of miR-590-5p/PELI1 axis in AD. The previous study has demonstrated that PELI1 exerts pivotal role in medicating microglial activation during the development of autoimmune encephalomyelitis through increasing the ubiquitination of tumor necrosis factor receptor-associated factor 3 (Traf3) and activating MAPK/P38 signaling pathway [28]. Similarly, this study also found that A*β*-induced BV-2 and HT22 cells exhibited decreased Traf3 expression and increased phosphorylation levels of ERK1/2 and P38, while the addition of miR-590-5p mimics or PELI1 silence partly reversed their expressions. Consistent with our results, activation of MAPK/P38 pathway is reported to be observed in the brain tissues of AD patients, and inhibiting MAPK/P38 signaling pathway conversely hampers the progression of AD [29, 30]. Therefore, we speculated that Traf3/MAPK P38 pathway participated in A*β*-induced BV-2 or HT22 cell injury.

## 5. Conclusion

In conclusion, our study showed that miR-590-5p was expressed at lower levels in AD, and miR-590-5p/PELI1 axis might be involved in A*β*-induced BV-2 or HT22 cell injury by the following Traf3/MAPK P38 pathway. These findings verified the involvement of miR-590-5p in the pathogenesis of AD based on animal and cell models.

## Figures and Tables

**Table 1:** Specific primers for qPCR assay.

Gene	Primer sequence
miR-590-5p	Sense primer: 5′-ACACTCCAGCTGGGGAGCTTATTCATAAAAGT-3′
	Antisense primer: 5′-CTCAACTGGTGTCGTGGA-3′
PELI1	Sense primer: 5′-GCCCCAGTAAAATATGGCGAA-3′
	Antisense primer: 5′-CCCCATTTGCCTTAGGTCTTT-3′
*β*-Actin	Sense primer: 5′-CATTGCTGACAGGATGCAGA-3′
	Antisense primer: 5′-AACGCTTCACGAATTTGCGT-3′
U6	Sense primer: 5′-CTCGCTTCGGCAGCACA-3′
	Antisense primer: 5′-AACGCTTCACGAATTTGCGT-3′

## Data Availability

All data generated or analyzed in this study are available in the published article.

## Additional Points

*Highlights*. (1) miR-590-5p was downregulated in AD. (2) PELI1 was proved as a target gene of miR-590-5p. (3) miR-590-5p/PELI1 axis was involved in A*β*-induced BV-2 or HT22 cell injury. (4) Traf3/MAPK P38 pathway participated in A*β*-induced BV-2 or HT22 cell injury.
